# The Treatment of Acute Gonorrhœa

**Published:** 1890-08

**Authors:** 


### THE TREATMENT OF ACUTE GONORRHŒA.

In the *Archiv fur Dermatologie und Syphilis* appears a paper by Dr. Friedheim, containing an elaborate account of a large number of experiments made in the clinic of Professor Neisser, of Breslau, with the view of determining the best method of treatment in acute gonorrhœa. The object aimed at was to find the local application which possessed in the highest degree the power (1) of killing the gonococci, (2) of influencing the inflammatory phenomena (3) of promoting epithelial desquamation, and thereby securing mechanical elimination of the micro-organisms. Among the numerous drugs experimented with were various preparations of mercury, including the perchloride and salicylate, permanganate of potash, iodoform, boric acid, pyrogallic acid, resorcin, antipyrin, thallin, and many others. But of all those now tried, the best results, as tested by the microscope, were obtained from a solution of nitrate of silver, of a strength varying from 1 in 4000 to 1 in 2000. The treatment is begun by the injection of this solution in the ordinary way, from four to six times a day, the result being that at first the discharge becomes more abundant, thicker, and more purulent ; but in about four days the secretion diminishes, becomes thin, and contains a quantity of epithelium. The gonococci also diminish in a remarkable manner, and after a few days disappear altogether. When this has taken place, the number of injections of nitrate of silver is reduced to two and afterwards to one daily ; and other injections, such as boric acid or some preparation of zinc, are used as well. But, in spite of the almost total disappearence of the discharge, the one daily injection of the nitrate of silver is to be kept up for many weeks. In cases where the nitrate of silver could not be borne, even in weaker solutions than those above mentioned, salicylate of mercury, or thallin, or the chloroborate of sodium was substituted with a certain amount of success ; and in the very rare cases in which no antibacterial injection could be tolerated, internal remedies were resorted to. Of these, cubebs, terpentine, oil of gaultheria, oil of sandal-wood, kaka-kaka, ichthyol, creolin, and copaiba were tried ; but among these, copaiba alone, and that only in some cases, was found to have any decided effect on the gonococci. As regards the danger of complications from the use of the nitrate of silver injections, it was found that this method of treatment was really the best preventive. Thus, among twelve hundred cases of gonorrhœa treated in various ways, there were one hundred and sixty-four of epididymitis, but in one hundred and forty-two of these the epididymitis was present when the patient first came under treatment; while of the remaining twenty-two cases, only one was being treated with nitrate of silver. The total number of cases given in the table which is appended to Dr. Friedheim's paper as having been treated by nitrate of silver injections is three hundred and eighteen, and thirty-seven of these it is stated that the antibacterial action of the drug was proved.—*British Medical Journal*, (*Therapeutic Gazette*).

---

BOSTON'S STREETS.—A petition, signed by over one hundred physicians of Boston, was recently submitted to the City Council, protesting against the filthy condition of the streets, and urging immediate remedy before the advent of hot weather. —*Journal Amer. Med. Association.*

